# The Effectiveness of Chlorhexidine and Air Polishing System in the Treatment of *Candida albicans* Infected Dental Implants: An Experimental In Vitro Study

**DOI:** 10.3390/antibiotics9040179

**Published:** 2020-04-14

**Authors:** Pier Carmine Passarelli, Marta De Leonardis, Giovan Battista Piccirillo, Viviana Desantis, Raffaele Papa, Edoardo Rella, Giuseppe Niccolò Mastandrea Bonaviri, Piero Papi, Giorgio Pompa, Guido Pasquantonio, Paolo Francesco Manicone, Antonio D’Addona

**Affiliations:** 1 Department of Head and Neck, Division of Oral Surgery and Implantology, Institute of Clinical Dentistry, Università Cattolica del Sacro Cuore, Fondazione Policlinico Universitario Gemelli, 00168 Rome, Italy; marta_de_leo@hotmail.it (M.D.L.); giovanbpiccirillo@gmail.com (G.B.P.); desantisviviana@gmail.com (V.D.); raffaele.papa@gmail.com (R.P.); rellaedoardo@gmail.com (E.R.); paolofrancesco.manicone@policlinicogemelli.it (P.F.M.); antonio.daddona@policlinicogemelli.it (A.D.); 2 Medicine and Surgery, Università Cattolica del Sacro Cuore, 00168 Rome, Italy; dott.mastandreabonaviri@gmail.com; 3 Department of Oral Surgery and Implantology, University of Rome La Sapienza, 00161 Rome, Italy; piero.papi@uniroma1.it (P.P.); giorgio.pompa@uniroma1.it (G.P.); 4 Department of Clinical Sciences and Translational Medicine, University of Rome Tor Vergata, 00133 Rome, Italy; guido.pasquantonio@uniroma2.it

**Keywords:** *Candida albicans*, chlorhexidine, dental implants, peri-implantitis

## Abstract

**Background:** Peri-implantitis is an inflammatory disease with an increasing diffusion rate which can affect the long-term survival of a prosthetic rehabilitation. The present study focused on the decontaminating efficacy of chlorhexidine and air polishing system with sodium bicarbonate powder against Candida albicans, a microorganism which seems to have a superinfecting opportunistic role in the pathology. The aim of the authors was to investigate and compare the effectiveness of these treatments, commonly used in clinical practice. Methods: An in vitro study was conducted to analyze the effects of two widely used therapeutic aids for the disinfection of affected titanium implants: chlorhexidine (CHX) and air polishing with sodium bicarbonate powder (P). A qualitative and quantitative comparative analysis of the residual biofilm was carried out using a colorimetric assay (XTT) and scanning electron microscopy (SEM) observation. The experiment was conducted both on machined titanium surfaces and on rough sandblasted ones with the aim of bringing out differences in the therapeutic outcomes concerning the superficial texture of the implant. The null hypothesis was that no difference could be detected between the samples, regarding both the treatments performed and the nano-structural features of titanium. **Results:** The best results (on both types of implant surfaces) were obtained when combining the use of chlorhexidine and air polishing (C + P). A linear decrease in the optical density (OD) values recorded at three different time points (30 s, 1 min, 5 min) was also observed passing from the first to the last one. When observed under scanning electron microscope rough surfaces showed an extensive and highly structured biofilm, more complex if compared to the one encountered when analyzing machined implants. Conclusions: the present pilot study showed that rough surfaces can promote fungal adhesion and eventually hinder the outcome of a decontaminating treatment. For this purpose, the physio-chemical technique is always more efficient if compared to a single-technique approach regardless of the surface characteristics.

## 1. Introduction

In modern dentistry, the use of titanium implants to restore edentulous areas of various extension is a very common practice [1,2,3], followed by a great percentage of long-term success [4]. Systemic or syndromic diseases [5,6] and immune disorders [7] should be evaluated before the implant placement in order to avoid post-operative bleeding-related complications [8,9] and to achieve better outcomes. An accurate exam of the bone crest, in terms of width and height, is also important for proper planning of the implant [10,11,12,13,14]. 

Once a dental implant is placed into the oral cavity its surface gets exposed to the oral environment, a very complex one characterized by a huge variety of resident microorganisms. The biodiversity of the oral microflora (over 600 different microbial species can be counted) and its balance with the host’s immune system is one of the defining factors of both health and well-being of peri-implant tissues [15]. 

Peri-implantitis is an inflammatory disease with bacterial etiology which affects the periodontal tissues surrounding a dental implant, accompanied by the progressive loss of supporting bone [16]. If not actively treated this pathology can lead to implant failure because of loss of osseointegration due to bone resorption [17]. 

Different studies have demonstrated that the subgingival microflora located in a periodontal pocket surrounding a titanium implant is far more complex compared to the one rediscovered in periodontitis [18] and that teeth and implants seem not to share the same microbiome, even in healthy conditions [19]. In fact, titanium can be easily colonized also by opportunistic pathogens which are not usually part of the oral microflora such as *Staphylococcus aureus*, *Pseudomonas aeruginosa*, Enterobacteriaceae and *Candida albicans* [20,21]. 

A small number of authors have focused their attention on the effective role of these superinfecting pathogens within the peri-implant disease. It is believed that they can sustain and worsen post-surgical oral infections when present in subgingival pockets together with the putative periodontopathogenic bacteria. They are usually associated with aggressive and recalcitrant forms of peri-implantitis, resistant to the therapeutic agents commonly used, besides being potential sources of systemic infections, especially dangerous for immunocompromised patients [22]. Even though their etiopathogenetic role needs to be further investigated, their virulence factors could suggest a possible involvement in the pathogenesis of peri-implantitis, especially with regards to *Candida albicans* [23]. 

This fungus can play a role in the development and progression of the disease because of its capacity of colonizing epithelial surfaces, inhibiting the function of polymorphonuclear neutrophil and secretion of lytic enzymes like collagenases, phospholipase, and proteinases. They can facilitate the penetration into the epithelial cell, the invasion of the gingival connective tissue, and the degradation of the immunoglobulins [24]. Furthermore, *Candida albicans* can induce inflammatory reactions and co-aggregate to different microorganisms, especially various species of streptococci and *Fusobacterium nucleatum*, contributing to the formation of multispecies biofilms [25]. They bind together throughout recognition receptor polysaccharides on the bacterial surface [26]. The simultaneous presence of microorganisms involved in the pathogenesis of peri-implantitis seems to upregulate its hyphal production and the expression of hydrolytic enzymes and genes associated with adhesion (ALS1 and ALS3) [27]. *Candida albicans* was found in the outer layer of subgingival biofilms associated to cases with poor response to the conventional treatment. Here it seemed to have a protective role in favor of the microorganisms of the deep interior from the action of immune mechanisms, thus aiding the resistance of the subgingival microbiota and contributing to the persistence of inflammation [28]. 

The treatment of peri-implantitis consists of the mechanical debridement of the infected metallic fixture to eradicate the pathological biofilm. The irregular rough surface of fixtures, highly micro and macro-structured to improve osseointegration, facilitates initial microbial adhesion and the formation of biofilms and makes sufficient debridement of implant surface very difficult to achieve [29,30]. Clinicians follow a combined protocol with the use of a variety of antimicrobial agents as adjunctive instruments helping to enhance and sustain the outcomes of surgical therapy [15,16,17]. Both mechanical (implantoplasty, air powder abrasive systems, laser and photodynamic therapy) and chemical agents (chlorhexidine, citric acid, hydrogen peroxide, antimicrobials) have been evaluated but still there is no evidence about what agent or technique should be used in the clinical practice for its superior efficacy [31,32,33].

The aim of the present study is to evaluate and compare the decontaminating properties of two of the most commonly used therapeutic agents in the treatment of peri-implantitis against *Candida albicans*: chlorhexidine (CHX) and air polishing with sodium bicarbonate powder used alone and then combined. The evaluation was conducted both on smooth and rough (sandblasted and acid etched) implants to highlight eventual differences in the outcomes related to the type of surface.

## 2. Results

Regarding machined implants, the sample used as a control showed the most intense coloration in the colorimetric assay (XTT) assay whereas the other experimental groups showed a color of decreasing intensity throughout the different time points: from a light orange observed at 30 s to no color recorded after 5 min (see optical density (OD) values in Table 1). 

**Figure 1:**
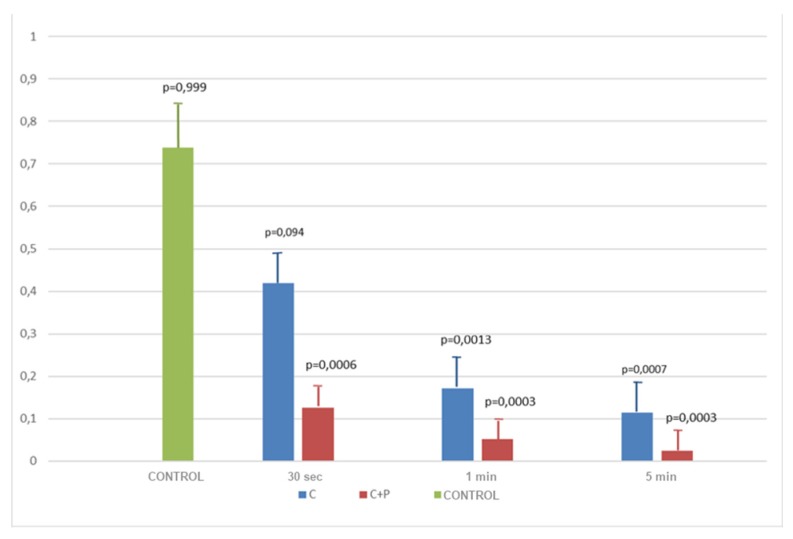
Efficacy of treatments on machined samples. Abbreviations: C = chlorhexidine; C + P = chlorhexidine + air polishing.

For a visual analysis of the results obtained on machined implants see Figure 1: the comparative histogram in the picture shows the mean efficacy of both treatments at each time point if compared to the control group and also reveals the reduction of the OD values recorded with the increasing of the time spent undergoing both types of treatment. In the end, considering the differences between the two therapeutic strategies, the combined one appeared to always be more effective rather than the simple use of CHX. 

**Figure 2:**
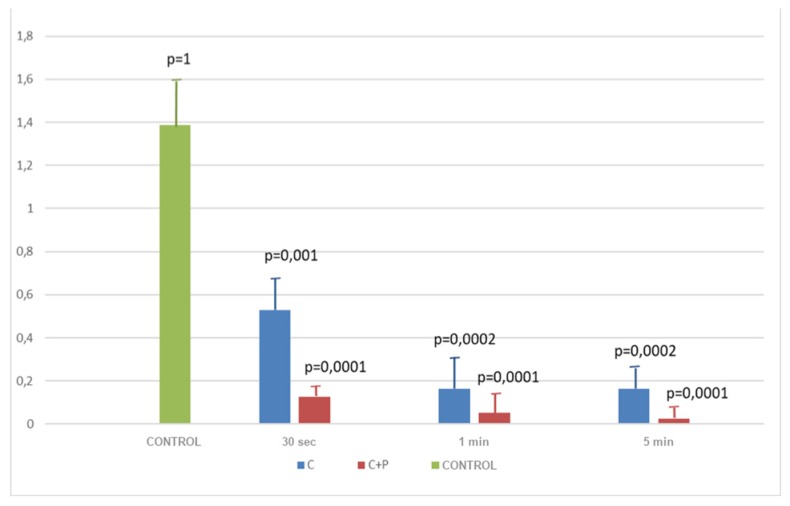
Efficacy of treatments on rough samples. Abbreviations: C = chlorhexidine; C + P = chlorhexidine + air polishing.

The same affirmation can be made with regards to rough implants. In fact, the histogram of Figure 2 shows the same decrease of the OD values related with the increase of time spent decontaminating the surfaces and the better efficacy of the double approach at each time point.

The most significant *p*-values were recorded at 5 min with the combined treatment (*p* = 0.0001 vs. *p* = 0.0002 obtained with CHX alone). 

In conclusion, it is possible to state that the best decontamination on both types of surfaces was obtained combining the use of air polishing and chlorhexidine. In fact, all *p*-values obtained from the comparison were significant (see Table 2 and Table 3).

**Figure 3:**
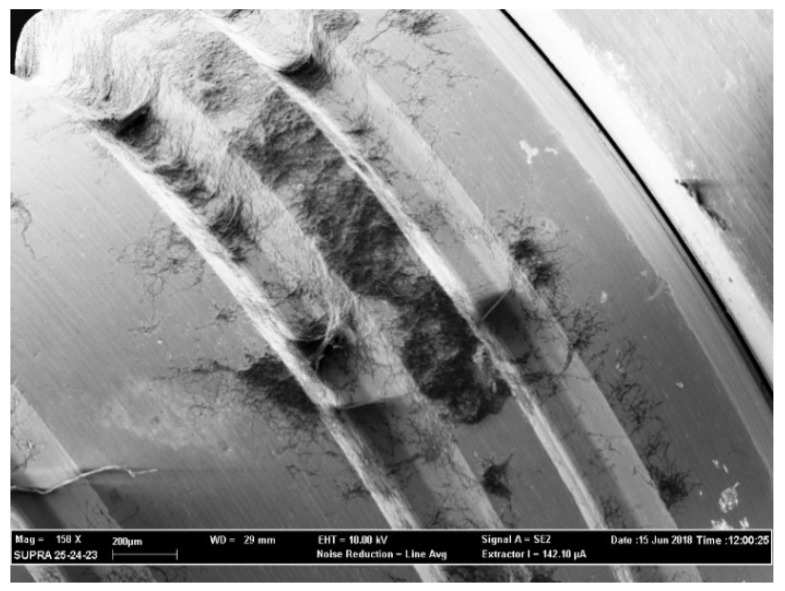
Chlorhexidine (CHX) treatment on a machined implant. Magnification 158×.

**Figure 4:**
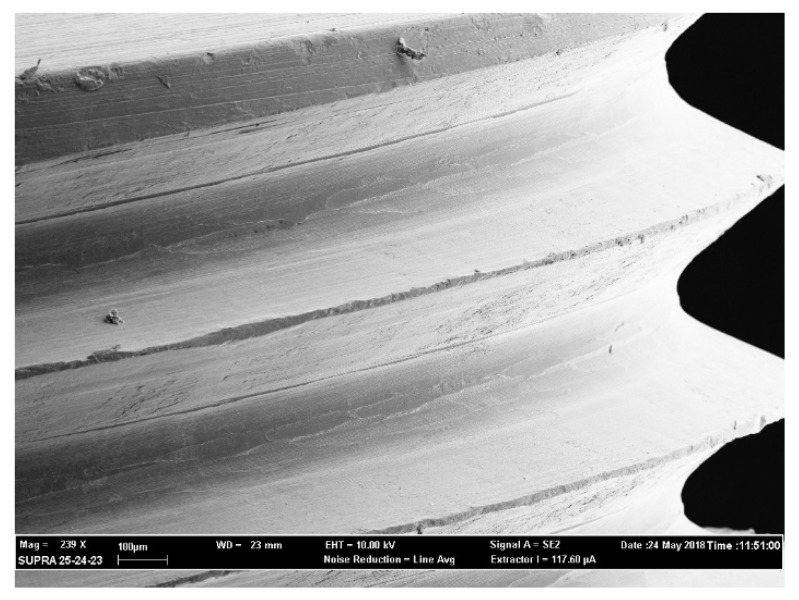
Air polishing and CHX treatment on a machined implant. Magnification 239×.

**Figure 5:**
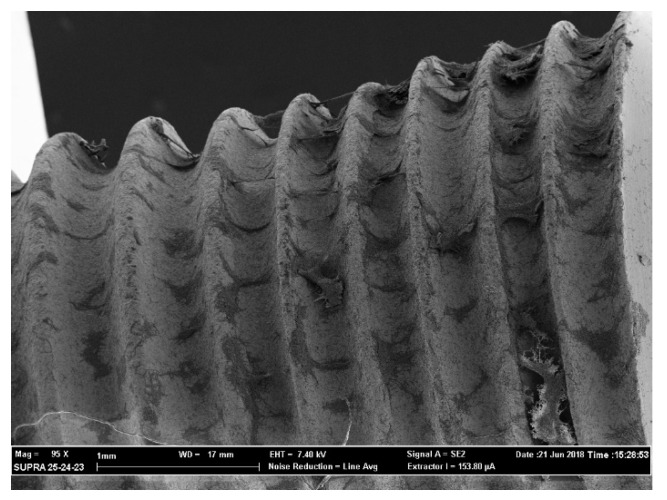
CHX treatment on a sandblasted implant. Magnification 95×.

**Figure 6:**
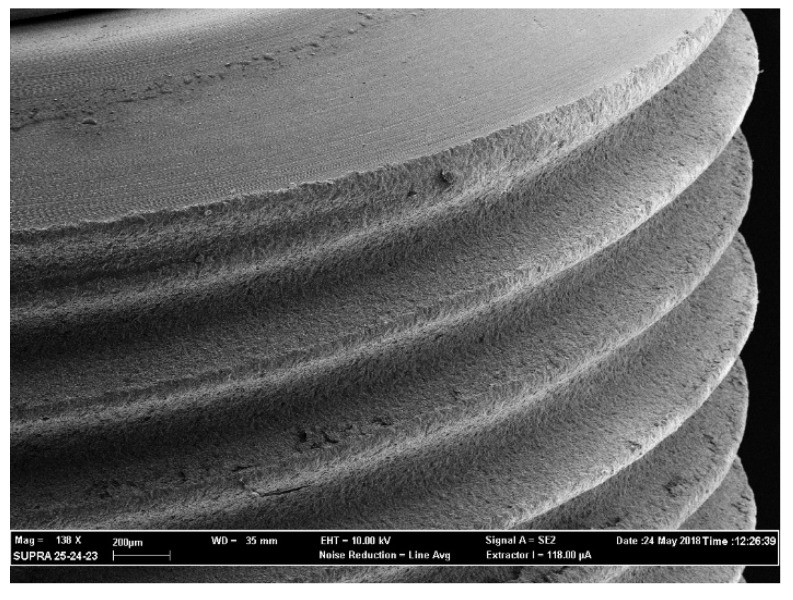
Air polishing and CHX treatment on a sandblasted implant. Magnification 138×.

The above-mentioned outcomes were confirmed during the observation of the samples with the use of a scanning electron microscope (SEM). From Figure 3, Figure 4, Figure 5 and Figure 6 it is easy to have an immediate visualization of the major effectiveness of the combined treatment protocol on both types of surfaces if compared to the use of CHX alone: in fact Figure 3 and Figure 5 show the presence of a residual well-structured biofilm which was not disrupted, whereas Figure 4 and Figure 6 show completely decontaminated fixtures. Comparing these images, it is also possible to visualize how surface roughness promotes the adhesion and the formation of a complex and more extended biofilm giving microorganisms a better adhesive substratum rather than a smooth one, which happened to be even more easily treatable. As far as surface characteristics are concerned, it is evident how this element plays a major role both in the contamination and the decontamination phase (see Table 2 and Table 3), confirmed by the OD values recorded in the control groups: the ones registered on the rough control are doubled when compared to the machined control ones whereas the OD values recorded after the C + P treatment on the sandblasted implants were higher than the ones recorded on the machined samples. 

## 3. Discussion

The present study focused on the efficacy of two treatment commonly used for the eradication of microbial biofilms from machined and rough titanium infected fixtures affected by peri-implantitis [34,35,36]. 

*Candida albicans* single-species biofilms were chosen because of their opportunistic role in the development of peri-implant disease and participation in the composition of sub-gingival peri-implant microflora [22,23,24]. Although frequently isolated as part of the peri-implant microflora, the etiopathogenetic role of *Candida albicans* within peri-implantitis is still unknown and needs further investigations but the microorganism’s virulence factors suggest its possible involvement in the pathogenetic process of the disease as they could sustain the progression of peri-implantitis [23]. 

Through this study, it was possible to demonstrate *Candida albicans*’ affinity for biomaterials, especially for titanium [37,38]. The persistence of pathogenic microorganisms adhering to implant surfaces is a clinically relevant problem, not just for the health of surrounding tissue and the implant, but also for the health of immunocompromised hosts.

The same attention to the control of active infection is needed when treating patients affected also by cardiovascular diseases (CVD): it has been demonstrated how high serum levels of c-reactive protein (CRP) are major predictors for high serum and salivary levels of endothelin 1 (ET-1). ET-1 plays a key role in the maintenance of homeostasis of endothelium thus having a possible correlation with the onset of CV events [39].

In these patients, microorganisms organized in a biofilm, which are more resistant to host defenses and conventional antimicrobial therapies, could spread through the ulcerate epithelium of the peri-implant pocket and become a source of persistent infections [37,38,40]. That is why dental implants covered by a pathogenic biofilm may be a source of recalcitrant infection and potential reservoirs of reinfection. 

Both treatments (CHX and C + P) were active against the fungus, confirming the findings of previous studies on their clinical utility [40,41,42,43,44] but comparing the OD values for the two treatments and *p*-values obtained from the statistical analysis it is evident how the air polishing system has the most relevant decontaminating power if compared to the use of CHX alone [45,46]. It mechanically disrupts the biofilm and so CHX does not seem to add any microbiological benefit to it, although always maintaining its clinical efficacy thanks to its antimicrobial properties [47]. 

Their combination resulted in a better strategy for the management of pathogenic biofilms with the possibility of overcoming the limitations of the chemical treatment alone, especially in the case of irregular surfaces, here empowering the outcome of a decontaminating treatment.

In the specific case of *Candida albicans*, the small irregularities of the sandblasted surface furnish the microorganism with protective niches from shear forces due to their dimensions (which are similar to the ones of the microorganism) and favor the biofilm formation [38]. 

## 4. Materials and Methods 

We tested 14 titanium implants measuring 5.2 mm in diameter and 10 mm long: seven implants presented a smooth machined surface whereas the other seven implants were rough sandblasted and acid etched. All implants were NQ implants and belonged to the UniQo line provided by Falappa Medical Devices (FMD, Rome, Italy).

### 4.1. In Vitro Biofilm Formation

*Candida albicans* clinical strain HBF6001 (taken from the internal storage of the Institute of Microbiology and Virology, Foundation Agostino Gemelli University Policlinic) was inoculated in 5 mL RPMI 1640 (ThermoFischer Scientific, Waltham, MA, USA) and incubated at 37 °C for 24 h on a shaking incubator (Iris Analytical Ltd, Camberley, England) at 150 r.p.m. (rotations per minute) (after 24 h cells usually reach a concentration of 1 × 10^8^ cells per mL). Then a 1:100 dilution of the original inoculum was made as follows: 13.5 mL RPMI 1640, 1.5 mL FCS (Fetal Calf Serum Sigma-Aldrich, St Louis, MI, USA), 150 μL inoculum, 15 μL gentamicin (Sigma-Aldrich, St Louis, Missouri, USA), and 15 μL vancomycin (Sigma-Aldrich, St Louis, Missouri, USA). Antibiotics were used to avoid bacterial growth in the solution and fetal calf serum to induce the expression of genes coding for virulence factors. 

The solution was kept in incubator on a shaking plate at 37 °C for 2 h and 30 min until an optical density of 0.7 read at 600 nm wavelength was reached, in order to have an approximative concentration of 1 × 10^7^ cells per mL. The solution was centrifuged at 4000 r.p.m. for 5 min and, after having discarded the supernatant, the cell pellet was resuspended in 15 mL of the same culture medium as before (13.5 mL RPMI 1640, 1.5 mL FCS, 15 μL gentamycin, and 15 μL vancomycin). The 14 implants were immersed into the solution and kept in a shaking incubator at 30 °C for 48 h. The culture medium was refreshed after 48 h after rinsing each implant with PBS (phosphate buffered saline Sigma-Aldrich, St Louis, Missouri, USA), to remove the cells loosely adherent to the implant surface but not really organized in a biofilm. After that, all the implants were placed again in a shaking incubator at 30 °C. The culture medium was refreshed after 48 h for a total incubation period of 1 week to guarantee the fungus the ideal condition for the gene expression of its virulence factors implied in the adhesion phase to the substratum. 

### 4.2. Implant Surface Decontamination

All implants were rinsed with sterile water for injections. One machined and one rough implant were used as a control, so they did not undergo any decontamination. They were kept first in saline (NaCl 0.9%) and then placed in formaldehyde 2%. The 12 remaining implants were located into six experimental groups, each composed of two specimens:

- Group 1: one machined and one rough implant treated with chlorhexidine 0.2% (Curasept ADS 0.2%, Curaden HealthCare Spa, Saronno, Italy) for 30 s.
- Group 2: one machined and one rough implant first treated with air polishing with sodium bicarbonate powder and then immersed in chlorhexidine 0.2% for 30 s.
- Group 3: one machined and one rough implant treated with chlorhexidine 0.2% for 1 min.
- Group 4: one machined and one rough implant first treated with air polishing with sodium bicarbonate powder and then immersed in chlorhexidine 0.2% for 1 min.
- Group 5: one machined and one rough implant treated with chlorhexidine 0.2% for 5 min.
- Group 6: one machined and one rough implant first treated with air polishing with sodium bicarbonate powder and then immersed in chlorhexidine 0.2% for 5 min.

All implants were treated by the same operator with Prophyflex III^®^ 2018 (KaVo, Biberach, Germany), under a pressure range between 3.2 and 5 bar. The nozzle was always kept at a distance of 3–5 mm from the implant surface with an angulation of 60–90° with the long axis of the implant. Each implant was instrumented for 1 min using regular movements of the nozzle along the surface of the fixture trying not to stop for more than 10 s on the same spot. 

### 4.3. Quantification of Biofilms

Biofilms on each implant surface were quantified using XTT (ThermoFischer Scientific, Waltham, Massachusetts, USA) assay, performed after drying all the samples with sterile gauzes. First, 10 mL of XTT was activated adding 10 μL of menadione and the total volume was divided for the 14 samples. XTT (2,3-bis (2-methoxy-4-nitro-5-sulfophenyl)-5-[(phenylamino) carbonyl]-2H-tetrazolium hydroxide) is a colorimetric assay used for the quantification of the vitality of the fungal cells of a biofilm. The assay is based on the transformation of XTT, a colorless tetrazolium salt, into water-soluble formazan, which has an orange dye ranging from light to bright. The assay shows a direct correlation between the number of viable cells and the intensity of the color obtained: the higher the intensity of the color, the higher the quantity of ATP contained in the solution, and so the number of viable cells adherent to the surface with metabolic activity, being dehydrogenases and reductases mitochondrial enzymes responsible for the chemical reaction. Each test should include a blank containing complete medium without cells used as a background control in order to have a comparison among the OD values recorded from the samples with the spectrophotometer. The samples were kept in the dark in a CO_2_ incubator at 37° and the results observed after 2 h. Finally, 300 μL of the solution where each sample was immersed was used for the recording of their OD by means of a spectrophotometer (Artiglass srl, Padova, Italy)

The optical density of each sample was read in triplicate, dividing 300 μL in three wells containing 100 μL each, and at a wavelength of 490 nm using the OD of the inactivated XTT as the 0 value (BLK). After recording the values samples were kept in formaldehyde 2% for 24 h to avoid the degradation of the organic parts of the biofilm. XTT analysis and SEM observation were used in association to evaluate the extension and the morphology of biofilms [48,49]. XTT has a high sensitivity for the quantification of cells’ viability and the data recorded were then confirmed by the images observed with the SEM analysis (see Figure 3, Figure 4, Figure 5 and Figure 6). 

### 4.4. SEM Observation

All samples were then rinsed with sterile water for injections and progressively dehydrated by immersion in 70% alcohol and 90% pure alcohol for 45 min each. Once dried all implants were mounted onto metallic stubs and metalized by electrodeposition of Au/Pa 60/40. The entire surface of each sample was observed, and images were taken at 50× and 100× magnification. Then, additional magnification of 1000× and 2000× was used to discover the details of biofilms particularly structured and largely extended on the metallic surface. This entire observational phase was conducted using a scanning electron microscope (SEM Supra 25, Carl Zeiss, Germany) held by the Department of Physics of the Catholic University of the Sacred Heart. 

### 4.5. Statistical Analysis

Statistical analysis was undertaken by an independent statistician with IBM SPSS Statistics software v.25 (IBM; Chicago, IL, USA). *p*-values were calculated for each group through parametric Student’s *t*-test and results with *p* < 0.05 were recorded as significant. 

## 5. Conclusions

The present in vitro study showed the affinity of *Candida albicans* for titanium and the differences in terms of colonization between smooth and rough surfaces. 

Dental implants can become a good substratum for fungal biofilm formation. Yeast cells have a high potential to adhere to artificial materials and the properties of the surface (i.e., roughness) influence the quantity and quality of fungal adhesion. Roughness provides a better osseointegration but enhances bacterial and fungal colonization and represents a very difficult device to decontaminate.

The results obtained from this study confirm the effectiveness of the airflow as a decontaminant instrument in the case of an infected fixture if compared to the use of CHX alone and the superiority of the combined treatment. This finding might be useful for a more efficient therapeutic approach against well-structured biofilms which are notoriously more resistant to conventional antimicrobial agents, not only with the aim of preserving the functionality of the rehabilitation but also for protecting and keeping the systemic health status of our patients. 

The present study should be considered as a pilot study and its results might be used as a basis for further investigations conducted on multi-species biofilms. 

## Figures and Tables

**Table 1:** Optical density (OD) values of all samples.

**Machined Implants**	**Treatment**	**Timing**	**Optical Density**			**Mean**	**SD**	***p*-Value**
	*CHX*	30 s	0.407	0.419	0.045	0.419	0.045	0.0094
	*CHX*	1 min	0.088	0.171	0.051	0.171	0.051	0.0013
	*CHX*	5 min	0.124	0.113	0.041	0.113	0.041	0.0007
	*C + P*	30 s	0.103	0.126	0.016	0.126	0.016	0.0006
	*C + P*	1 min	0.043	0.052	0.011	0.051	0.011	0.0003
	*C + P*	5 min	0.017	0.024	0.014	0.023	0.014	0.0003
	*Control*	-	0.826	0.738	0.084	0.738	0.084	0.999
**Rough Implants**	*CHX*	30 s	0.543	0.471	0.573	0.529	0.043	0.001
	*CHX*	1 min	0.205	0.17	0.144	0.163	0.037	0.0002
	*CHX*	5 min	0.188	0.158	0.142	0.162	0.019	0.0002
	*C + P*	30 s	0.045	0.041	0.043	0.042	0.001	0.0001
	*C + P*	1 min	0.035	0.014	0.018	0.043	0.012	0.0001
	*C + P*	5 min	0.035	0.059	0.033	0.022	0.009	0.0001
	*Control*	-	1.563	1.356	1.239	1.386	0.133	1
**BLK**	-	-	0	−0.001	0.002	-	-	-

Differences in the color intensity were also recorded between the types of treatment. Although both treatments determined a significant reduction in terms of OD at 5 min compared to the control sample (*p*-value CHX at 5 min = 0.0007; *p*-value C + P at 5 min = 0.0001), the use of air polishing (C + P) gave more promising results rather than chlorhexidine (CHX) used alone for all samples exposed to it (*p*-value CHX vs. C + P = 0.0173). The most striking difference between the treatments can be observed at the first time point (30 s): the use of air polishing allows obtaining a 4–5 points reduction in the OD values recorded with the use of CHX alone, both on machined and rough implants. Abbreviations: CHX = chlorhexidine; C + P = chlorhexidine + air polishing; BLK = blank, 0 value.

**Table 2:** Comparative table for smooth surfaces.

Surface	Timing	Treatment	Mean	*p*-Value
Machined	30 s	CHX	0.419	0.001
Machined	30 s	C + P	0.126	
Machined	1 min	CHX	0.114	0.1054
Machined	1 min	C + P	0.052	
Machined	5 min	CHX	0.172	0.0173
Machined	5 min	C + P	0.024	

**Table 3:** Comparative table for rough surfaces.

Surface	Timing	Treatment	Mean	*p*-Value
Rough	30 s	CHX	0.529	<0.0001
Rough	30 s	C + P	0.043	
Rough	1 min	CHX	0.163	0.0067
Rough	1 min	C + P	0.022	
Rough	5 min	CHX	0.163	0.0016
Rough	5 min	C + P	0.042	
